# Circulating miRNA is a useful diagnostic biomarker for nonalcoholic steatohepatitis in nonalcoholic fatty liver disease

**DOI:** 10.1038/s41598-021-94115-6

**Published:** 2021-07-19

**Authors:** Tae Hyung Kim, Yoonseok Lee, Young-Sun Lee, Jeong-An Gim, Eunjung Ko, Sun Young Yim, Young Kul Jung, SeongHee Kang, Moon Young Kim, Hayeon Kim, Baek-hui Kim, Ji Hoon Kim, Yeon Seok Seo, Hyung Joon Yim, Jong Eun Yeon, Soon Ho Um, Kwan Soo Byun

**Affiliations:** 1 Department of Internal Medicine, Division of Gastroenterology and Hepatology, Guro Hospital, Korea University College of Medicine, Korea University Medical Center, 97, Guro-Dong Gil, Guro-Dong, Guro-Ku, Seoul, 08308 Republic of Korea; 2 Medical Science Research Center, Korea University Medical Center, Anam-dong 5-ga, Seongbuk-gu, Seoul, 02841 Republic of Korea; 3 Department of Internal Medicine, Wonju Severance Christian Hospital, Wonju, Republic of Korea; 4 Department of Pathology, Korea University Medical Center, Seoul, Republic of Korea

**Keywords:** Gastroenterology, Hepatology, Diagnostic markers

## Abstract

Nonalcoholic steatohepatitis (NASH) is considered as a progressive form of nonalcoholic fatty liver disease (NAFLD). To distinguish NASH from nonalcoholic fatty liver (NAFL), we evaluated the diagnostic value of circulating miRNAs. Small RNA sequencing was performed on 12 NAFL patients and 12 NASH patients, and the miRNA expression was compared. After selecting miRNAs for the diagnosis of NASH, we analyzed the diagnostic accuracy of each miRNA and the combination of miRNAs. External validation was performed using quantitative reverse transcription PCR. Among the 2,588 miRNAs, 26 miRNAs significantly increased in the NASH group than in the NAFL group. Among the 26 elevated miRNAs in the NASH group, 8 miRNAs were selected, and in silico analysis was performed. Only four miRNAs (miR-21-5p, miR-151a-3p, miR-192-5p, and miR-4449) showed significant area under the receiver operating characteristic curve (AUC) values for NASH diagnosis. The combination of the four miRNAs showed satisfactory diagnostic accuracy for NASH (AUC 0.875; 95% CI 0.676–0.973). External validation revealed similar diagnostic accuracy for NASH (AUC 0.874; 95% CI 0.724–0.960). NASH represents significantly distinct miRNA expression profile compared with NAFL. The combination of serum circulating miRNAs can be used as a novel biomarker for the NASH diagnosis in NAFLD.

## Introduction

As prevalence of nonalcoholic fatty liver disease (NAFLD) is increasing, it has become a major cause of chronic liver disease^1,2^. Nonalcoholic steatohepatitis (NASH) is considered a progressive disease of NAFLD, which is distinguished from non-alcoholic fatty liver (NAFL)^3^. NASH is an important factor for disease progression of NAFLD and the diagnosis of NASH requires histologic confirmation with steatosis, inflammation, and hepatocyte ballooning appearance^4,5^. Therefore, a liver biopsy is essential to confirm NASH, but has several limitations such as high cost, complication risks, and sampling errors^6^. Developing noninvasive tools for evaluating NAFLD severity is an emerging interest of hepatologists, especially for the discrimination of NASH or advanced fibrosis among NAFLD^7^. Several serologic and imaging biomarkers have been developed and validated for detecting advanced fibrosis such as the Fibrosis 4 index, NAFLD fibrosis score, transient elastography (TE), and magnetic resonance elastography (MRE)^8^. For noninvasive diagnosis of NASH, several serologic markers were investigated^7^, but the diagnostic accuracy of those markers was limited. Among them, cytokeratin 18 (CK18) levels are elevated during the apoptosis or necroptosis of hepatocytes in patients with NASH, and CK18 is a well-validated single marker for the diagnosis of NASH^9^. CK18 exhibited moderate diagnostic accuracy for NASH in a meta-analysis, but the cut-off for NASH diagnosis is variable, and its availability is limited^10^. Therefore, there is an urgent need to develop more accurate biomarkers for the diagnosis of NASH.

MicroRNA (miRNA) is a small-sized non-coding RNA comprising 20–25 nucleotides, and it binds to the target mRNA, resulting in translation inhibition or mRNA degradation^11,12^. miRNAs are synthesized in a variety of cells and participate in cell signaling^13^; they have important roles in regulating cell growth, proliferation, and metabolism^14^. In addition to their physiological role, circulating miRNAs have been studied as a candidate for diagnosis of a variety of diseases such as malignancy and cardiovascular, neurologic, and metabolic diseases, including diabetes and NAFLD^15,16^. In patients and animal models with NAFLD, circulating miRNAs exhibit significant differences compared with healthy controls^17,18^. Circulating miRNA-34a, miRNA-122, and miRNA192 consistently increase in patients with NASH compared to patients with simple steatosis^19^. Diagnostic accuracy to distinguish NASH from NAFL was evaluated using miR-34a, but it showed moderate accuracy [area under the receiver operating characteristic curve (AUC) = 0.78)].

In the present study, we aimed to develop a biomarker to diagnose NASH in NAFLD by analyzing the circulating miRNA expression profile in sera from patients with biopsy-confirmed NAFLD using emerging next generation sequencing (NGS).

## Results

### Baseline characteristics

For miRNA sequencing, sera from 24 biopsy-proven patients with NAFLD were collected between February 2019 and July 2019, including 12 patients with NAFL and 12 patients with NASH. The baseline characteristics of patients are summarized in Table 1. The NASH group was more women dominant (75%) and showed lower body mass index (BMI), hemoglobin, total bilirubin, albumin, and creatinine compared with the NAFL group. Steatosis, lobular inflammation, and fibrosis did not show significant differences in severity between the NAFL and NASH groups. Ballooning is essential for the diagnosis of NASH; there was a significant difference in the presence of ballooning between the NAFL and NASH groups. The NASH group showed significantly higher NAFLD activity score (NAS) than that observed for the NAFL group. Representative histopathological findings are presented in Supplementary Fig. 1. In the validation cohort, 37 patients with biopsy-confirmed NAFLD were enrolled, including 11 and 26 patients with NAFL and NASH, respectively (Supplementary Table 1). Clinical and laboratory characteristics were similar in both groups, except for aspartate aminotransferase (AST). We observed no significant differences for steatosis, lobular inflammation, and fibrosis between the NAFL and NASH groups in the validation cohort; however, hepatocyte ballooning and NAS were found to be significantly higher in the NASH group compared with those in the NAFL group in the validation cohort.

**Table 1:** Baseline characteristics.

Characteristics	NAFL (n = 12)	NASH (n = 12)	*P-*value
Age, median (IQR), years	46.5 (41.75–52.50)	51 (44.5–64)	0.165
Male/female	8/4 (66.7/33.3)	3/9 (25.0/75.0)	0.013
DM, no. (%)	7 (58.3)	11 (91.7)	0.059
HTN, no. (%)	7 (58.3)	7 (58.3)	1.000
Dyslipidemia, no. (%)	4 (33.3)	5 (41.7)	0.673
BMI, median (IQR), kg/m^2^	32.30 (29.14–35.06)	27.30 (26.89–31.04)	0.015
Hb, median (IQR), g/dL	15.05 (13.63–16.03)	13.2 (12.9–13.8)	0.002
PLT, median (IQR), X 10^3^/μL	231 (181–258)	221.5 (192–288)	0.624
AST, median (IQR), IU/L	44 (36–109)	75.5 (51–128)	0.194
ALT, median (IQR), IU/L	75 (38–164)	112 (40–202)	0.665
ALP, median (IQR), IU/L	74 (57–82)	80 (69–87)	0.259
GGT,median (IQR), IU/L	68 (35–105)	75 (33–120)	0.908
Bilirubin,median (IQR),mg/dL	0.70 (0.53–1.18)	0.37 (0.24–0.53)	0.005
Albumin, median (IQR), g/dL	4.4 (4.2–4.6)	4.2 (4.0–4.3)	0.019
PT, median (IQR), INR	0.98 (0.95–1.04)	0.97 (0.93–1.01)	0.247
Creatinine (IQR), mg/dL	0.82 (0.66–0.85)	0.61 (0.55–0.71)	0.009
CRP (mg/L)	1.73 (0.80–3.79)	3.23 (0.97–4.74)	0.419
Histological finding*			
Steatosis score, n (%) 0/1/2/3	0 (0.0%)/4 (33.3%)/ 5 (41.7%)/3 (25.0%)	0 (0.0%)/3 (25.0%)/ 5 (41.7%)/4 (33.3%)	0.867
Lobular inflammation score, n (%) 0/1/2/3	0 (0.0%)/7 (58.3%)/ 4 (33.3%)/1 (8.3%)	0 (0.0%)/3 (25.0%)/ 8 (66.7%)/ 1 (8.3%)	0.231
Ballooning score, n (%) 0/1/2	12 (100.0%)/0 (0.0%)/0 (0.0%)	0 (0.0%)/ 7 (58.3%)/5 (41.7%)	< 0.001
NAFLD activity score n (%) 2/3/4/5/6/7/8	2 (16.7%)/6 (50.0%)/2(16.7%)/1 (8.3%)/1 (8.3%)/0 (0.0%)/0 (0.0%)	0 (0.0%)/1 (8.3%)/0 (0.0%)/6 (50.0%)/4 (33.3%)/1 (8.3%)/0 (0.0%)	0.016
Fibrosis stage, n (%) 0/1/2/3/4	6 (50.0%)/2 (16.7%)/ 2 (16.7%)/1 (8.3%)/ 1 (8.3%)	3 (25.0%)/3 (25.0%)/ 3 (25.0%)/3 (25.0%)/ 0 (0%)	0.493

*NAFL* nonalcoholic fatty liver, *NASH* nonalcoholic steatohepatitis, *IQR* interquartile range, *BMI* body mass index, *DM* diabetes mellitus, *HTN* hypertension, *Hb* hemoglobin, *PLT* platelet, *AST* aspartate transaminase, *ALT* alanine transaminase, *ALP* alkaline phosphatase, *PT* prothrombin time, *INT* international normalized ratio, *GGT* gamma glutamyl transferase, *CRP* C-reactive protein.

*Histological findings were analyzed based on NAFLD activity scores developed by NASH clinical research network.

### miRNA expression profiles

**Figure 1:**
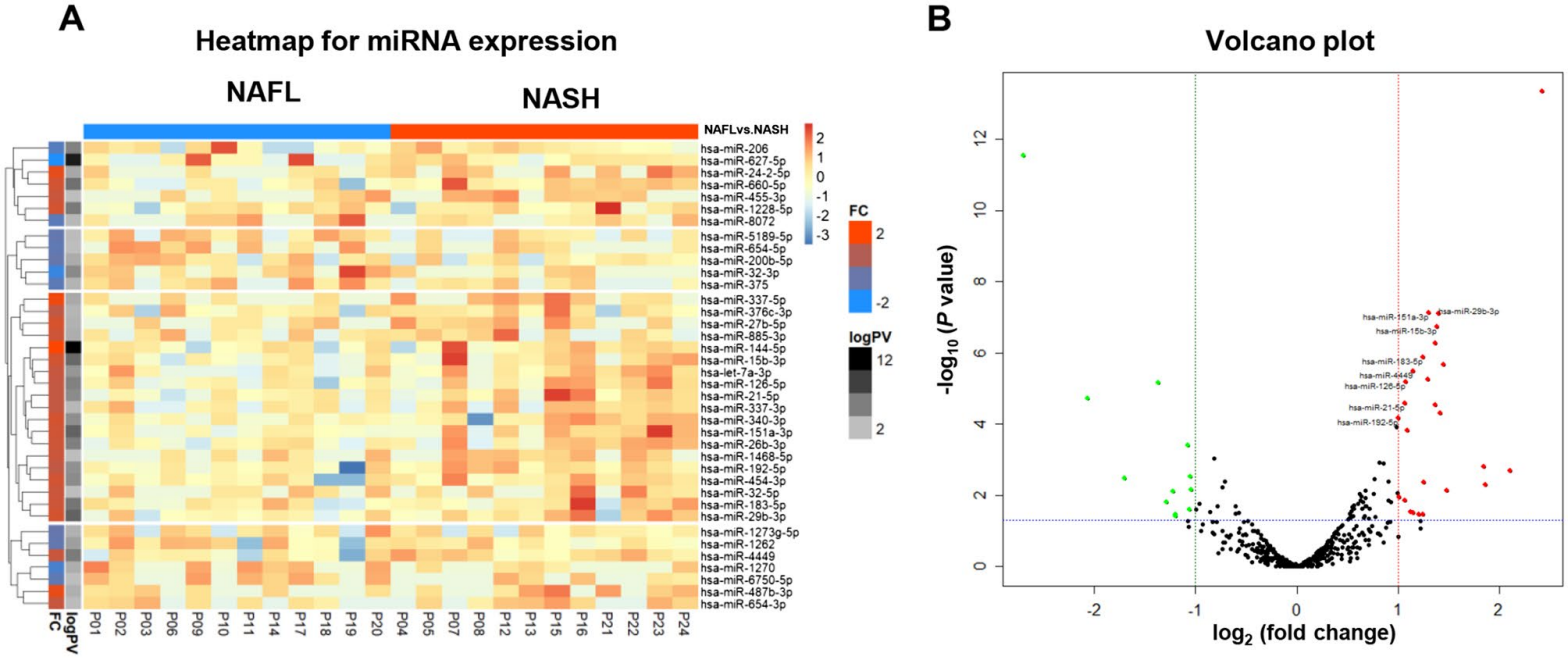
Heatmap (**A**) and volcano plot (**B**) of miRNA expression profile in sera of patients with NAFL and NASH. In heatmap, column annotation bar indicates two groups of disease (NAFL and NASH), and two row annotation bars indicate *P*-value and fold change between two groups. In row annotation bars, black and red color indicate high significance and higher expressed in NASH. Green dots indicate miRNA with decreased expression [log_2_(fold change) − 1] in NASH group (*P* < 0.05). Red dots indicate miRNA with increased expression [log_2_(fold change) > 1] in NASH group (*P* < 0.05). The dashed blue line represents where *P* < 0.05 and the threshold for high statistical significance.

In miRNA analysis, a total of 2588 miRNAs were analyzed, and each sample expressed 332 to 618 miRNAs (median 469 miRNAs). Approximately 2153 miRNAs were excluded from analysis because these miRNAs were expressed in < 50% samples; therefore, 435 miRNAs were compared between NAFL and NASH groups (Supplementary Fig. 2). A total of 38 miRNAs showed a significant difference in expression between the NAFL group and NASH group as shown in the heatmap (Fig. 1A). Twenty-six miRNAs significantly increased more than two times in the NASH group as compared to the NAFL group (*P* < 0.05) (Fig. 1B). In contrast, 12 miRNAs significantly decreased in the NASH group as compared to the NAFL group (*P* < 0.05).

### Diagnostic accuracy of miRNAs for NASH

**Figure 2:**
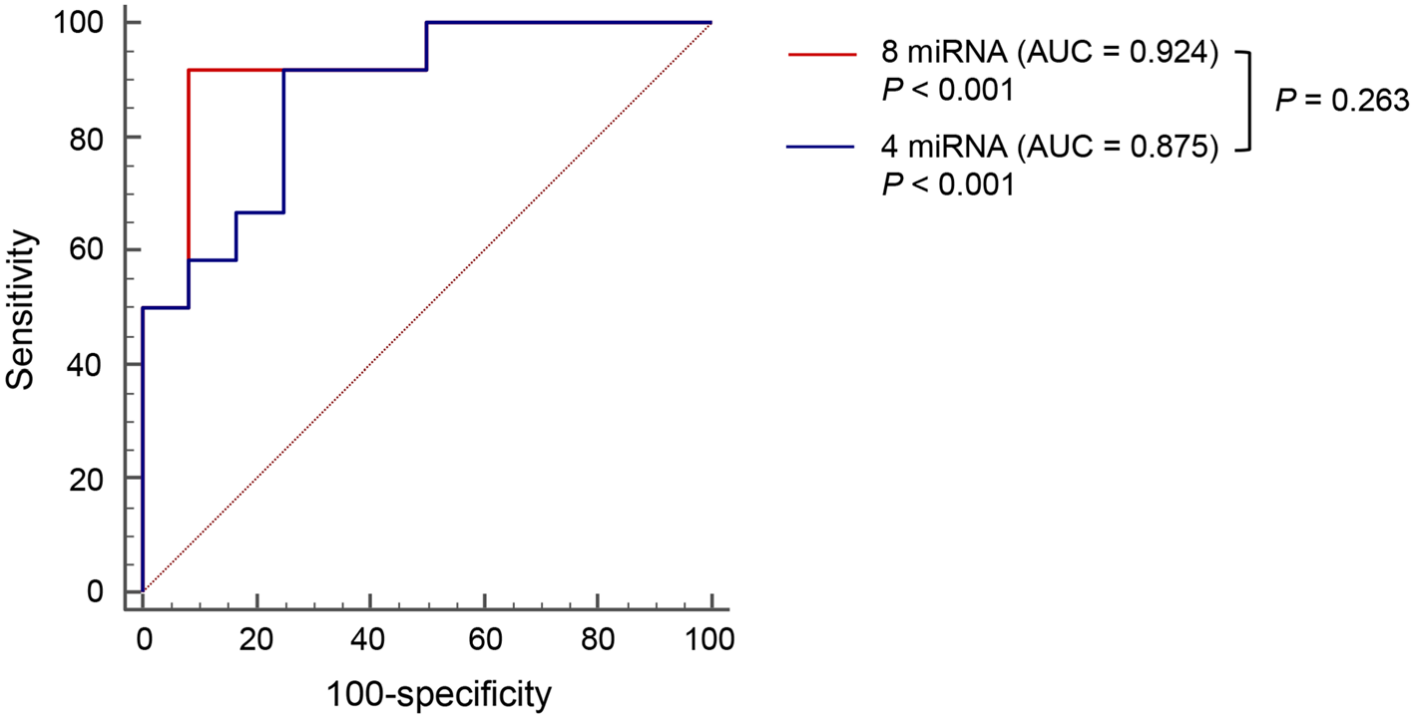
Diagnostic accuracy of miRNA combination. Receiver operating characteristic (ROC) curves for diagnostic accuracy for NASH using miRNA expression level from miRNA sequencing. The red line represents the ROC curve for a combination of eight miRNAs (miR-15b-3p, miR-21-5p, miR-29b-3p, miR-126-5p, miR-151a-3p, miR-183-5p, miR-192-5p, and miR-4449). The blue line represents the ROC curve for the combination of four miRNAs (miR-21-5p, miR-151a-3p, miR-192-5p, and miR-4449).

Eight miRNAs (miR-15b-3p, miR-21-5p, miR-29b-3p, miR-126-5p, miR-151a-3p, miR-183-5p, miR-192-5p, and miR-4449) were selected to evaluate the diagnostic accuracy for NASH. These eight miRNAs increased more than 2 times in the NASH group compared with the NAFL group and showed upper 25% normalized expression value in the NASH group among 435 miRNAs that were expressed in > 50% of 24 samples (Supplementary Fig. 2). Because 12 miRNAs that significantly increased in the NAFL group compared with the NASH group showed low detection as normalized expression values, these 12 miRNAs were excluded from further analysis. The ROC curve and comparison of normalized expression value for miRNAs between NAFL and NASH groups are shown (Supplementary Fig. 3). The AUC, sensitivity, and specificity of eight miRNAs are summarized in Table 2. Only four miRNAs (miR-21-5p, miR-151a-3p, miR-192-5p, and miR-4449) showed meaningful AUC values for NASH diagnosis (*P* < 0.05). When AUC values for diagnosis of NASH were compared between a combination of eight miRNAs (miR-15b-3p, miR-21-5p, miR-29b-3p, miR-126-5p, miR-151a-3p, miR-183-5p, miR-192-5p, and miR-4449) (AUC, 0.924; 95% CI 0.739–0.992) and a combination of four miRNAs (miR-21-5p, miR-151a-3p, miR-192-5p, and miR-4449) (AUC, 0.875; 95% CI 0.676–0.973), there was no significant difference (*P* = 0.263) (Fig. 2).

**Table 2:** Diagnostic accuracy of miRNA for NASH from NGS analysis.

miRNA	Normalized expression value				AUC	*P-*value (AUC)	Sensitivity	Specificity
	NAFL (n = 12)	NASH (n = 12)	*P-*value	Cut-off value				
miR-15b-3p	6.96 ± 0.33	7.90 ± 0.45	0.00002	8.36	0.667 (0.447–0.844)	0.154	41.67	100
miR-21-5p	8.01 ± 0.22	8.79 ± 0.33	0.00081	8.93	0.736 (0.518–0.893)	0.032	58.33	100
miR-29b-3p	7.24 ± 0.38	8.33 ± 0.52	< 0.00001	8.21	0.694 (0.475–0.864)	0.089	58.33	83.33
miR-126-5p	12.93 ± 0.33	13.84 ± 0.39	0.00024	14.05	0.694 (0.475–0.864)	0.084	50.00	91.67
miR-151a-3p	11.39 ± 0.16	12.37 ± 0.34	< 0.00001	12.35	0.750 (0.533–0.902)	0.030	66.67	100
miR-183-5p	7.57 ± 0.39	8.29 ± 0.49	0.00008	7.95	0.618 (0.399–0.807)	0.322	66.67	58.33
miR-192-5p	7.97 ± 0.45	9.15 ± 0.27	0.00173	7.95	0.771 (0.555–0.916)	0.007	100	50.00
miR-4449	8.64 ± 0.58	10.28 ± 0.33	0.00015	9.67	0.743 (0.525–0.898)	0.018	66.67	75.00
Combination of 8 miRNAs					0.924 (0.739–0.992)	< 0.001	91.67	91.67
Combination of 4 miRNAs					0.875 (0.676–0.973)	< 0.001	91.67	75.00

*NASH* nonalcoholic steatohepatitis, *NAFL* nonalcoholic fatty liver, *AUC* area under the receiver operating characteristic curve. *P*-value means that.

We also compared miRNA expression between patients with and without fibrosis to identify the confounding effect of fibrosis in NASH (Supplementary Fig. 4). Although 14 miRNAs showed significant differences in expression, there was no overlap between these 14 miRNAs and 8 miRNAs that were selected to evaluate the diagnostic accuracy for NASH in Supplementary Fig. 2.

### External validation by comparing miRNA expression using qRT-PCR between NAFL and NASH

**Figure 3:**
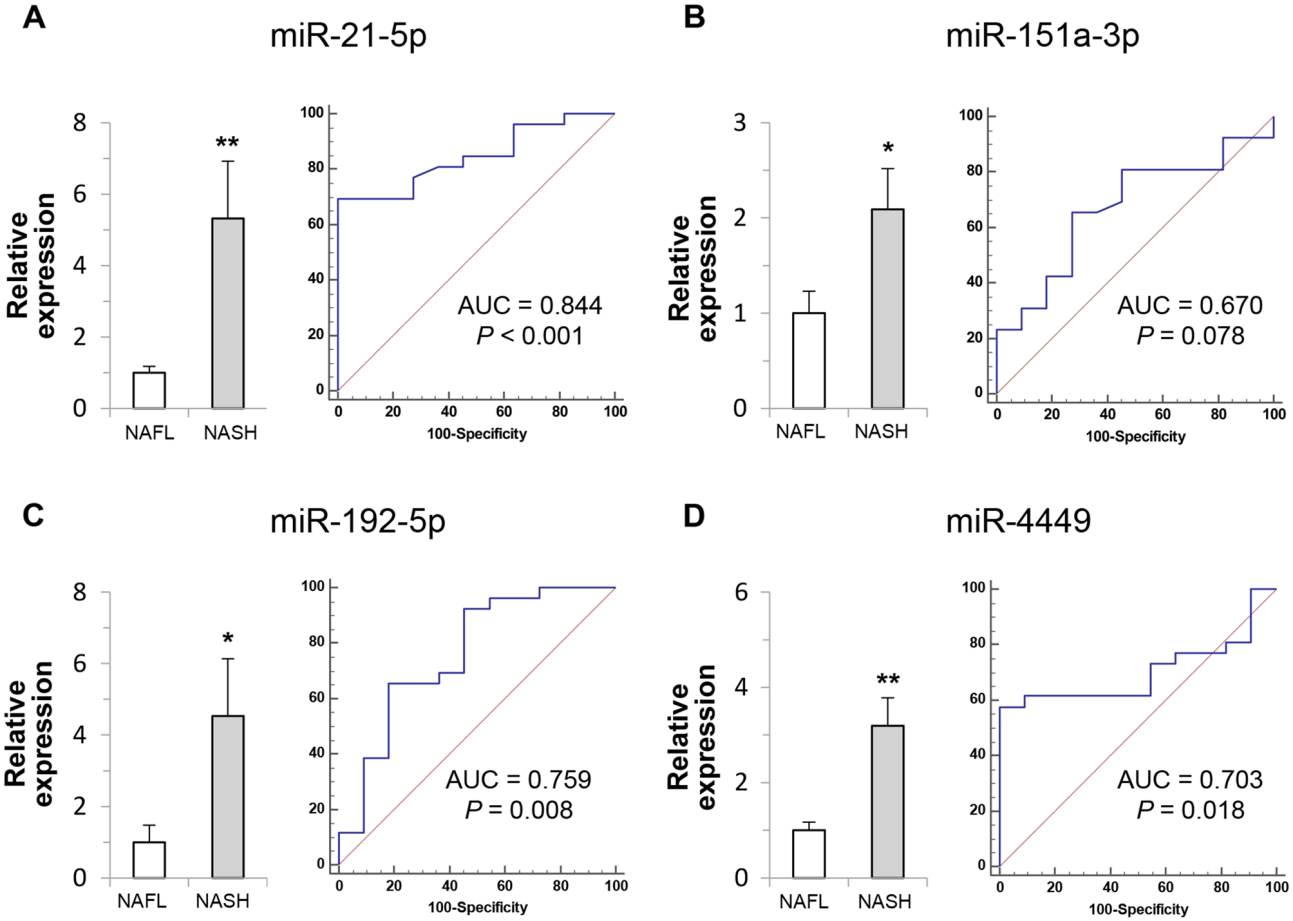
Expression level and diagnostic receiver operating characteristic (ROC) curve for NASH diagnosis of four miRNAs (miR-21-5p, miR-151a-3p, miR-192-5p, and miR-4449) in validation cohort. Expression level of each miRNA is normalized to small nuclear RNA U6 expression value and represents 2^-∆∆Ct^. *Indicates *P* < 0.05 compared with the corresponding control. **Indicates* P* < 0.01 compared with the corresponding control.

**Figure 4:**
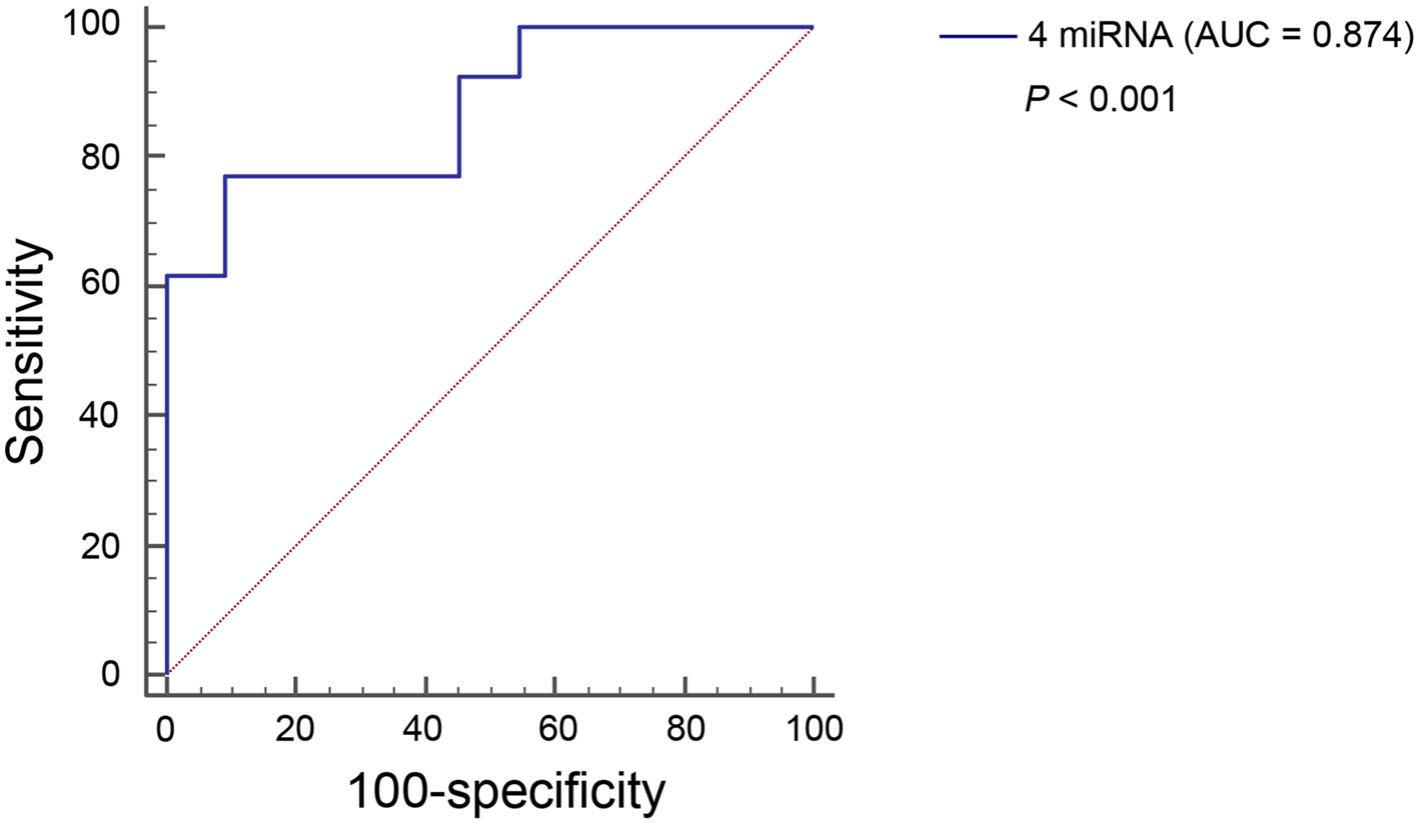
Diagnostic accuracy of miRNA combination in validation cohort. Receiver operating characteristic (ROC) curve for diagnostic accuracy for NASH in validation cohort. The blue line represents the ROC curve for the combination of four miRNAs (miR-21-5p, miR-151a-3p, miR-192-5p, and miR-4449) with ∆∆Ct value.

We analyzed the expression of circulating miRNAs between NAFL and NASH in an external validation cohort from another tertiary center. The expression of miR-21-5p, miR-151a-3p, miR-192-5p, and miR-4449 was higher in the NASH group than in the NAFL group (Fig. 3). For the combination of miR-21-5p, miR-151a-3p, miR-192-5p, and miR-4449, the AUC value for diagnosis of NASH was 0.874 (95% CI 0.724–0.960, *P* < 0.001) (Fig. 4).

### miRNA-mRNA correlation and Kyoto Encyclopedia of Genes and Genomes (KEGG)

We compared eight miRNAs (miR-15b-3p, miR-21-5p, miR-29b-3p, miR-126-5p, miR-151a-3p, miR-183-5p, miR-192-5p, and miR-4449) with upregulated expression and 265 genes with downregulated expression in the NASH group studied previously as GSE48452^20^. We confirmed 26 pairs of miRNA**-**mRNA interactions that were predicted by mirDIP^21^ and integrated in a single network constructed by Cytoscape^22^. In the predicted interactions, five miRNAs (miR-21-5p, miR-29b-3p, miR-126-5p, miR-183-5p, and miR-192-5p) were linked to 17 mRNAs (*CADM2*, *CTH*, *DNAJC12*, *GPAM*, *HSD17B11*, *IGF1*, *NAMPT*, *NIPAL1*, *PCDH20*, *PLSCR4*, *RBMS1*, *RNF152*, *SLC16A1*, *SLC16A10*, *SLC16A7*, *SLC19A2*, and *SOCS2*). According to the fold changes between NAFL and NASH, each node is indicated by color keys (Supplementary Fig. 5A).

Then, we confirmed enriched KEGG pathways detected from the eight highly expressed miRNAs in the NASH group. TGF-β and Wnt signaling pathways are depicted, and red nodes represent factors inhibited by eight miRNAs (Supplementary Fig. 5B). A total of 10 pathways were associated with four or more miRNAs (Supplementary Table 2).

## Discussion

As the need for noninvasive testing to determine the severity of NAFLD increases, various biomarkers have been investigated to diagnose NASH or advanced fibrosis. However, biomarkers for NASH that represent progressive inflammation of hepatocytes have shown limited accuracy and accessibility^7^. In this study, we found a combination of several circulating miRNAs using NGS and qRT-PCR, which could be a useful biomarker to diagnose NASH.

miRNA is a small non-coding RNA, comprising ≤ 25 nucleotides. The major role of miRNAs is post-transcriptional inhibition of gene expression by binding to the 3′-untranslated region of target mRNAs^23^. Circulating miRNAs are derived from specific cells and reflect the presence of disease or disease severity. The expression of miRNAs depends on the presence of NAFLD or the disease severity of NAFLD; therefore, miRNA expression has been suggested as a diagnostic biomarker for NAFLD, NASH, and advanced fibrosis^19^. Several circulating miRNAs, such as miR-16, miR-21-5p, miR-34a, miR-122, miR-192, and miR-375, have shown higher expression in the sera of patients with NASH than in those with NAFL. Most of these studies evaluated miRNA expression using real-time PCR quantification. More than 2600 sequences of human mature miRNAs are known in miRBase^24^; therefore, there is a limitation in evaluating the overall expression profile of whole human mature miRNAs. Our study analyzed a total of 2588 mature circulating miRNA expression profile using NGS. We selected four candidate miRNAs as biomarkers for NASH diagnosis and analyzed their diagnostic value using normalized values from NGS data. A combination of four miRNAs, miR-21-5p, miR-151a-3p, miR-192-5p, and miR-4449 showed significant diagnostic accuracy with an AUC of 0.875 in the normalized value from NGS data. According to external validation using qRT-PCR, we found that the diagnostic accuracy of a combination of miR-21-5p, miR-151a-3p, miR-192-5p, and miR-4449 was sufficient for them to be a biomarker for NASH diagnosis. Among human mature miRNAs, miR-122-5p is the major miRNA that is expressed in the liver, and circulating miR-122-5p increases in patients with NAFLD^25^. Although miR-122-5p was the second most abundant circulating miRNA in our study after miR-423-5p, the difference in expression level of circulating miR-122-5p between NAFL and NASH groups was not significant.

miR-21-5p increases in the plasma of patients with NASH^26^ and it is associated with hepatic metabolism, inflammation, and lipid metabolism^27,28^. miR-21-5p has been identified as a typical onco-miRNA in many previous studies. Our study indicated that miR-21-5p was highly expressed in NASH, and the relationship among miR-21-5p, NASH, and liver cancer could be an interesting topic for further study. Plasma level of miR-151a-3p is positively correlated with that of TNF-α, which is the classical inflammatory parameter and major factor in the progression of NAFLD^29^. Circulating miR-192-5p is also upregulated in patients with NASH as compared to patients with NAFL^17^. Exosomes from lipotoxic hepatocytes showed increased miR-192-5p, and exosomal miR-192-5p regulates disease progression of NAFLD by activating proinflammatory macrophages^30,31^. miR-4449 expression is rarely known in patients with NAFLD. In patients with obesity, circulating exosomal miR-4449 showed increased expression as compared to the healthy control group, and its expression was decreased after bariatric surgery^32^. The increasing patterns of our miRNAs exhibited consistency with other liver-related pathology states or obesity.

We applied a previous study uploaded in GEO to network analysis. From the GSE48452 dataset, we identified 265 downregulated genes in patients with NASH, and 26 pairs of miRNA–mRNA interactions were selected. Thus, bioinformatic analysis was applied to explore the correlation between miRNA–mRNA expression profiles. The target genes downregulated with upregulated miRNAs in NASH could be revealed by the public expression dataset.

This study has some limitations**.** First, the expression levels of miRNAs were standardized to U6 as an internal control. Although not all studies used snRNA U6 as internal control, many other studies used snRNA U6 as internal control^33–35^. Further validation studies are required to evaluate the possible clinical application of miRNAs as diagnostic biomarkers using other standardized control. Second, sequencing was conducted using a small number of patients. To overcome this limitation, we conducted external validation with patients from other centers, and we found similar diagnostic accuracy for NASH in an external validation cohort. However, further validation studies with larger populations and varying degrees of fibrosis are required. Third, we could only provide relative expression level of miRNA for the diagnosis of NASH in patients with NAFLD not absolute expression level. Further studies are required for clinical application.

In conclusion, NASH represents significant distinct miRNA expression profiles compared with NAFL. A combination of serum circulating miRNAs including miR-21-5p, miR-151a-3p, miR-192-5p, and miR-4449 could be used as a novel biomarker for the diagnosis of NASH in NAFLD.

### Patients and methods

#### Patients and sera collection

For small RNA sequencing, 24 patients with biopsy-confirmed NAFLD, comprising 12 NAFL patients and 12 NASH patients, were enrolled from our previous study at Korea University Guro Hospital^36^. Another 37 patients with biopsy-confirmed NAFLD from Anam Hospital were enrolled for comparison of miRNA expression between NAFL and NASH in external validation. Patients were excluded if they had consumed excessive alcohol and had viral hepatitis, autoimmune hepatitis, primary biliary cholangitis, decompensated cirrhosis, or other severe systemic diseases.

Laboratory tests were performed before liver biopsy, and sera were collected during blood sampling for laboratory tests. Sera were stored at -80℃ and thawed just before RNA extraction. This study had been approved by the institutional review board from Korea University Guro Hospital (2016GR0302) and Anam Hospital (2018AN0129). All patients agreed to the sera collection and submitted written informed consent. All investigators conducted this study in accordance with the Declaration of Helsinki. Each author reviewed and approved the final manuscript.

#### Liver biopsy and histopathological evaluation

NAFLD was diagnosed in all patients by percutaneous liver biopsy via intercostal spaces. Using an 18-gauge Tru-cut needle (TSK laboratory, Tochigi, Japan), two pieces of minimum 2-cm length were obtained from the specimens. Tissues were fixed in formalin, and paraffin tissue blocks were made. Tissue slides were made with 4-μm sections and performed hematoxylin & eosin and Masson’s trichrome staining. Histological findings were analyzed based on NAS developed by NASH clinical research network^37^. NAFL was defined when > 5% of hepatic steatosis was presented without hepatocyte ballooning, whereas NASH was defined when > 5% of hepatic steatosis and inflammation with hepatocyte ballooning were presented^4^.

#### Small RNA sequencing and analysis

Total circulating RNA from sera was seperated using the miRNeasy Serum/Plasma Kit (Qiagen, Valencia, CA) under the manufacture’s guidelines. The concentration of extracted RNA was determined using Quant-IT RiboGreen (Invitrogen, Carlsbad, CA, USA), and RNA size was measured using an Agilent RNA 6000 Pico Kit and Small RNA Kit on an Agilent 2100 Bioanalyzer (Agilent Technologies, Böblingen, Germany). For the construction of sequencing libraries, 10 ng of RNA was used with the SMARTersmRNA-Seq Kit for Illumina according to the manufacturer’s instructions. The purification of amplified libraries was performed using 6% Novex tris–borate-EDTA polyacrylamide gel electrophoresis gels (Thermo Fisher, MA, USA) from 171 to 253 bp (18 to 100 bp cDNA plus 153 bp of adaptors) fraction. After quantification with quantitative PCR (qPCR) of guidance with the Quantification Protocol Guide (KAPA Library Quantification kits for Illumina Sequencing platforms) and qualification with the TapeStation D1000 ScreenTape (Agilent Technologies, Waldbronn, Germany), the libraries sequencing was done by an Illumina HiSeq 2500 (Illumina, San Diego, CA, USA). To eliminate adapter sequences, the raw reads of small RNAs were processed using the Cutadapt program (NBIS, Uppsala, Sweden).

#### Quantitative reverse transcription PCR

cDNA synthesis was done using reverse transcriptase with miRNA-specific stem-loop primers (Applied Biosystems). qRT-PCR was performed on a QuantStudio 6 Flex Real-time PCR system (Applied Biosystems) using Taqman master mixture. The relative abundance of miRNA was normalized to that of small nuclear RNA U6. The relative amount of each miRNA was measured using the 2^−∆∆Ct^ method. The primers are summarized (Supplementary Table 3).

### mRNA data collection and analysis

Data mining from Gene Expression Omnibus database (GEO; https://www.ncbi.nlm.nisteatohepatitish.gov/geo/) was performed to confirm a spectrum of differentially expressed mRNA profiles of NASH. Gene microarray expression profiles between the NAFL group and NASH group were collected from GEO using the keyword “steatosis and steatohepatitis.” GSE48452 was suitable for our analysis and downloaded to select genes differently expressed in the NASH group. Differently expressed genes were retrieved by *t*-test using R software and filtered when log_2_(fold change) > 1 or < − 1, and *P* < 0.05 between the NAFL group and NASH group. An miRNA–mRNA network was constructed using mirDIP^21^ and visualized using Cytoscape version 3.8.0^22^.

### Statistics

The demographics and laboratory results are represented as percentage and median with first quartile to third quartile. Pearson’s chi-squared test for categorical variables and the Man–Whitney U-test for continuous variables were used to compare baseline characteristics. The cut-off and AUC values for the diagnosis of NASH were determined by receiver operating characteristic (ROC) curve analysis and based on histologic diagnosis. Logistic regression analysis was performed to combine miRNA value. For statistical analysis, SPSS (version 25; IBM Corporation, Armonk, NY, USA) and MedCalc (version 17.6; MedCalc Software, Ostend, Belgium) were used.

## Supplementary Information

Supplementary Information.
