# Supplementary material for: Circulating miRNA is a useful diagnostic biomarker for nonalcoholic steatohepatitis in nonalcoholic fatty liver disease
Source: Sci Rep. 2021 Jul 19;11:14639. doi: 10.1038/s41598-021-94115-6 (PMC8289842; doi:10.1038/s41598-021-94115-6)
Supplement: Supplementary file 1 — Supplementary Information. [file 41598_2021_94115_MOESM1_ESM.docx]

**Circulating miRNA is a useful diagnostic biomarker for nonalcoholic steatohepatitis in nonalcoholic fatty liver disease**

Tae Hyung Kim^1,†^, Yoonseok Lee^1, †^, Young-Sun Lee^1,*^, Jeong-An Gim^2,*^, Eunjung Ko^1^, Sun Young Yim^1^, Young Kul Jung^1^, SeongHee Kang^3^, Moon Young Kim^3^, Hayeon Kim^4^, Baek-hui Kim^4^, Ji Hoon Kim^1^, Yeon Seok Seo^1^, Hyung Joon Yim^1^, Jong Eun Yeon^1^, Soon Ho Um^1^, and Kwan Soo Byun^1^

^1^Department of Internal Medicine, Korea University Medical Center, Republic of Korea

^2^Medical Science Research Center, Korea University Medical Center, Republic of Korea

^3^Department of Internal Medicine, Wonju Severance Christian Hospital, Republic of Korea

^4^Department of Pathology, Korea University Medical Center, Republic of Korea

^†^ These authors contributed equally to this work as first author.

^*^These authors contributed equally to this work as corresponding author.


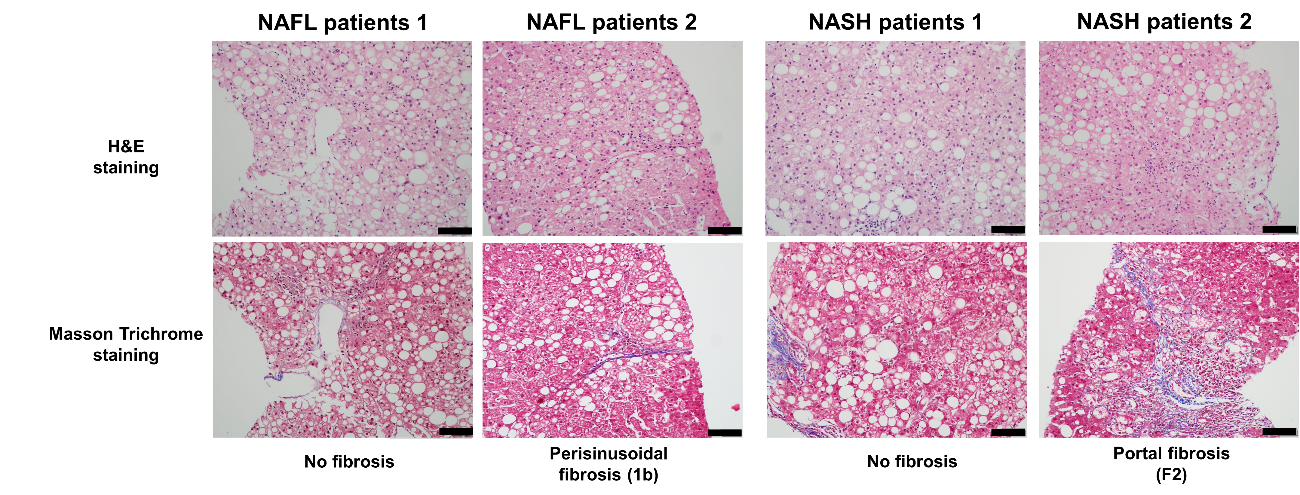


**Supplementary Figure 1.** Histopathological findings for NAFL and NASH groups. The bars in each micrograph represent 100μm.

NAFL, nonalcoholic fatty liver; NASH, nonalcoholic steatohepatitis; H&E, hematoxylin and eosin staining.

miRNA with significant difference of expression

(*n* = 38)

Abundant expression in NASH group

(*n* = 26)

miRNA with the top 25% expression in NASH group

(n = 8)

Expression ≥50% samples

(*n* = 435)

Total analyzed miRNA

(*n* = 2588)

**Supplementary Figure 2.** The selection process for miRNAs as a diagnostic biomarker for NASH.

**
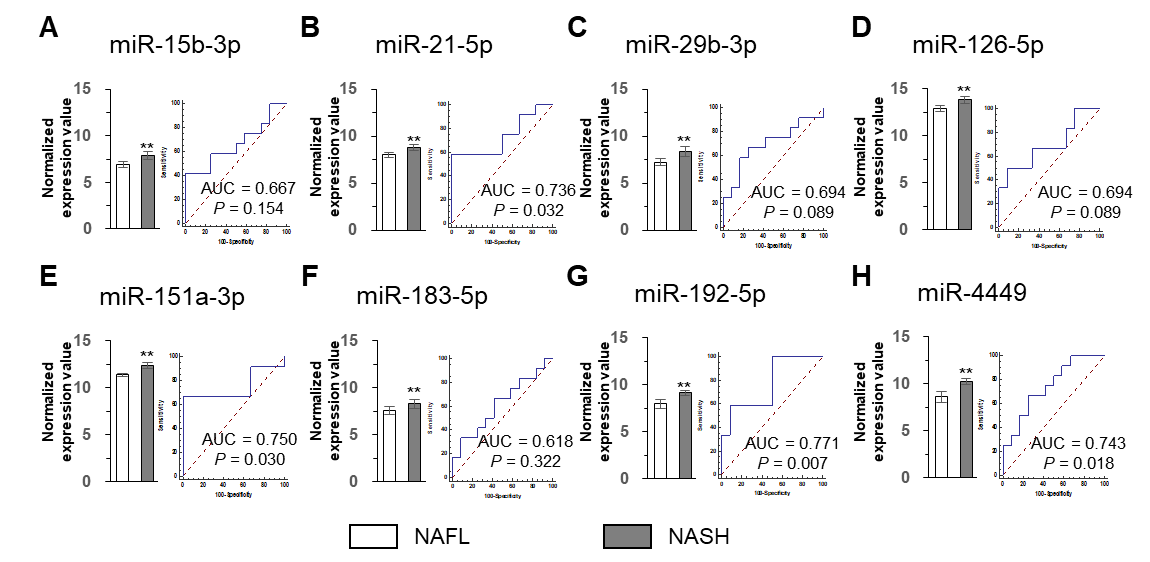
**

**Supplementary Figure 3. Expression level and diagnostic receiving operating characteristic (ROC) curve of eight miRNAs for NASH diagnosis.** The expression level of each miRNA represents normalized expression value.

^**^ indicates *P* < 0.01 compared with the corresponding control.


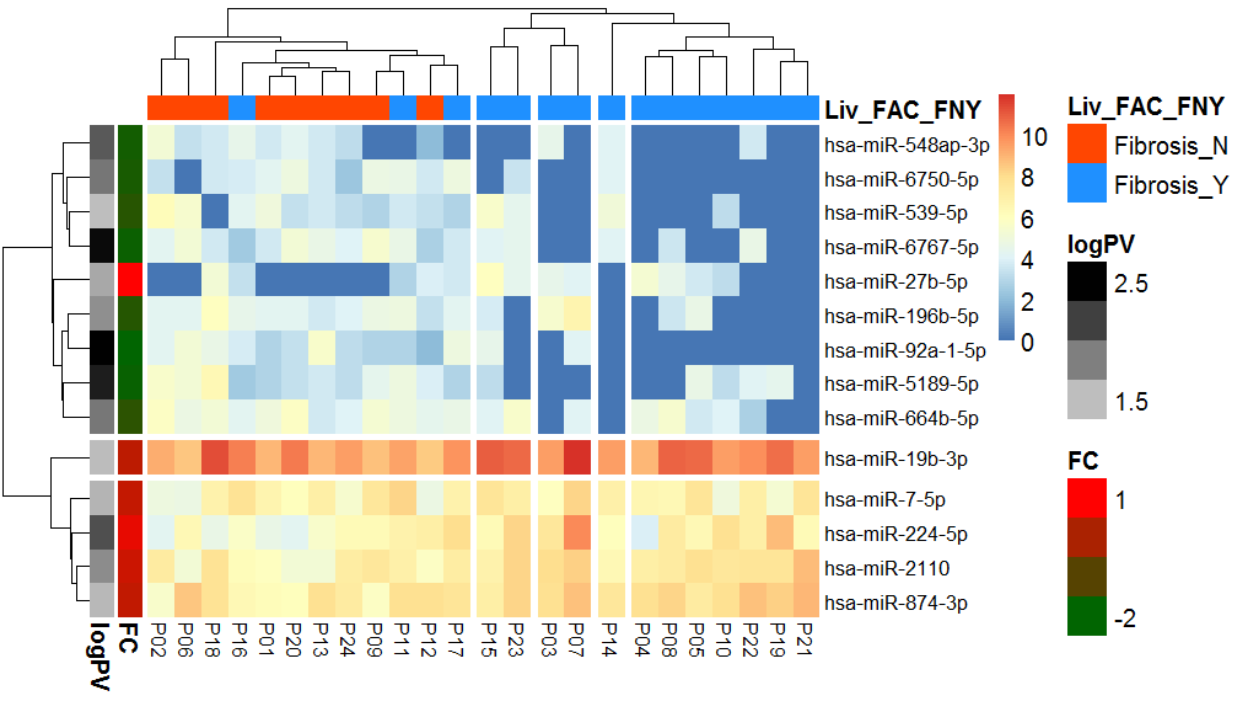


**Supplementary Figure 4.** Heatmap of miRNA expression profile in the sera of patients with and without fibrosis. Column annotation bar indicates two groups of disease (fibrosis and without fibrosis), and two-row annotation bars indicate *P*-value and fold change between two groups. In row annotation bars, black and red colors indicate the high significance and higher expression in the fibrosis group.

**
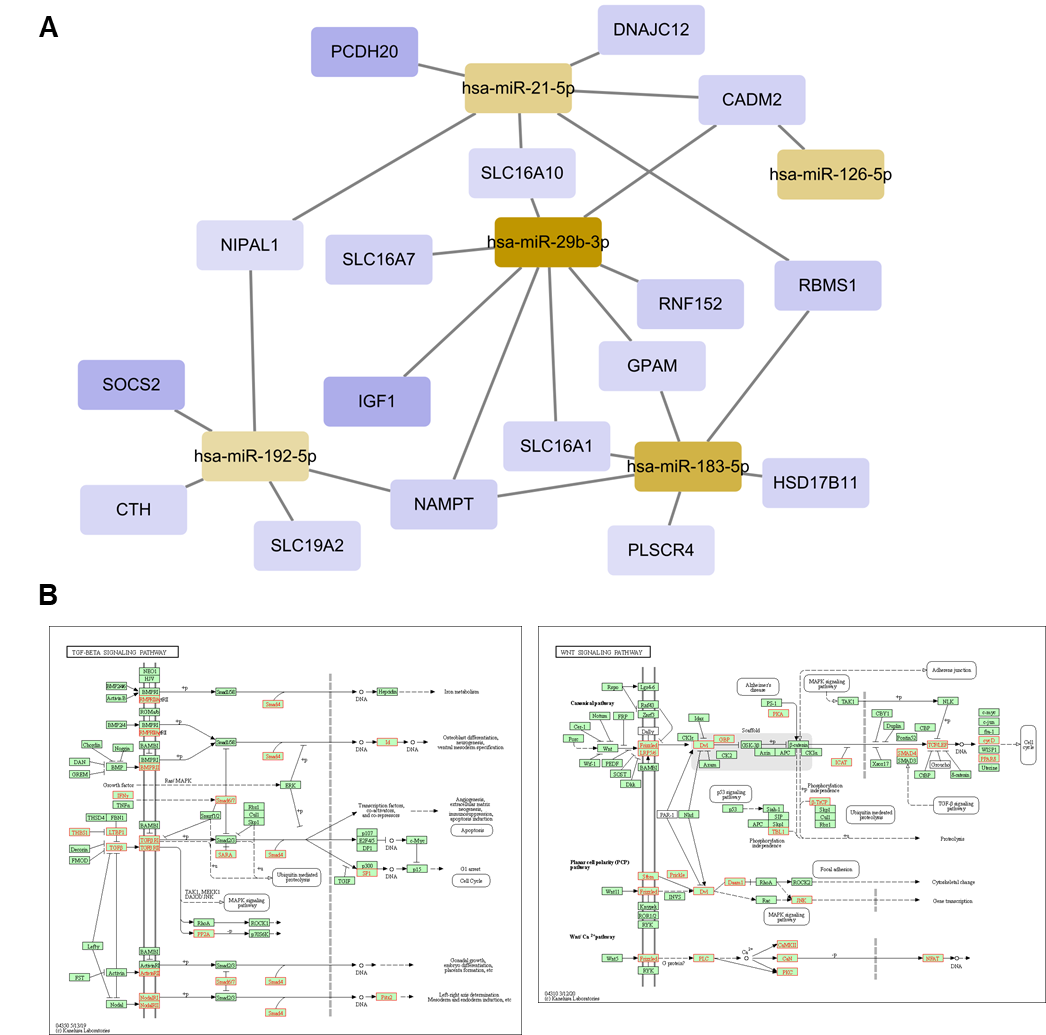
**

**Supplementary Figure 5.** Construction of miRNA–mRNA network. Global scheme of the network between patients with NAFL and patients with NASH are depicted (A). The ocherous color nodes denote highly expressed miRNAs, whereas the purple color nodes denote downregulated mRNAs in NASH. TGF-β and Wnt signaling pathways are indicated, and red nodes denote inhibited factors by eight miRNAs (B).

We received permission from Kanehisa Laboratories to use KEGG pathway map.

**Supplementary Table 1.** Baseline characteristics of validation cohort

| Characteristics | NAFL  (n = 11) | NASH  (n = 26) | *p-* value |
| --- | --- | --- | --- |
| Age, median (IQR), years | 41.0 (37.0–43.5) | 48.5 (35.0–58.0) | 0.200 |
| Male/Female | 9/2 (81.8/18.2) | 17/9 (65.4/34.6) | 0.544 |
| DM, No. (%) | 1 (9.1) | 9 (34.6) | 0.233 |
| HTN, No. (%) | 3 (27.3) | 12 (46.2) | 0.482 |
| Dyslipidemia, No. (%) | 3 (27.3) | 10 (38.5) | 0.783 |
| BMI, median (IQR), kg/m^2^ | 25.90 (25.15–30.05) | 29.65 (26.89–35.60) | 0.144 |
| Hb, median (IQR), g/dL | 14.70 (14.25–15.30) | 14.60 (13.60–15.20) | 0.752 |
| PLT, median (IQR), × 10^3^/μL | 280 (262–287) | 240 (215–281) | 0.056 |
| AST, median (IQR), IU/L | 29 (24–45) | 55 (33–72) | 0.029 |
| ALT, median (IQR), IU/L | 53 (44–82) | 94 (45–129) | 0.207 |
| ALP, median (IQR), IU/L | 75 (60–78) | 81 (72–90) | 0.096 |
| GGT, median (IQR), IU/L | 38 (34–45) | 43 (30–94) | 0.352 |
| Bilirubin, median (IQR), mg/dL | 0.90 (0.70–1.00) | 0.70 (0.60–0.90) | 0.202 |
| Albumin, median (IQR), g/dL | 4.4 (4.2–4.5) | 4.3 (4.0–4.4) | 0.399 |
| PT, median (IQR), INR | 0.91 (0.91–0.95) | 0.93 (0.91–0.98) | 0.472 |
| Creatinine (IQR), mg/dL | 0.87 (0.73–0.91) | 0.87 (0.69–1.00) | 0.702 |
| CRP (mg/L) | 1.40 (0.70–5.25) | 1.95 (1.10–3.70) | 0.653 |
| Histological finding^*^ |  |  |  |

| Steatosis score,  n (%) 0/1/2/3 | 0 (0%)/ 7 (63.6%)/  3 (27.3%)/ 1 (9.1%) | 0 (0%)/ 14 (53.8%)/  8 (30.8%)/ 4 (15.4%) | 0.823 |
| --- | --- | --- | --- |
| Lobular inflammation score,  n (%) 0/1/2/3 | 0 (0.0%)/ 8 (72.7%)/  3 (27.3%)/ 0 (0%) | 0 (0.0%)/ 22 (84.6%)/  4 (15.4%)/ 0 (0%) | 0.281 |
| Ballooning score,  n (%) 0/1/2 | 11 (100%)/  0 (0%)/ 0 (0%) | 0 (0%)/  22 (84.6%)/ 4 (15.4%) | < 0.001 |
| NAFLD activity score  n (%) 2/3/4/5/6/7/8 | 7 (63.6%)/ 0 (0%)/ 3 (27.3%)/ 1 (9.1%)/ 0 (0%)/ 0 (0%)/ 0 (0%) | 1 (3.8%)/ 13 (50.0%)/ 3 (11.5%)/7 (26.9%)/2 (7.7%)/ 0 (0%)/ 0 (0%) | 0.001 |
| Fibrosis stage,  n (%) 0/1/2/3/4 | 4 (36.4%)/ 7 (63.6%)/  0 (0%)/ 0 (0%)/ 0 (0%) | 6 (23.1%)/ 15 (57.7%)/  5 (19.2%)/ 0 (0%)/ 0 (0%) | 0.264 |

IQR, interquartile range; BMI, body mass index; DM, diabetes mellitus; HTN, hypertension; Hb, hemoglobin; PLT, platelet; AST, aspartate transaminase; ALT, alanine transaminase; ALP, alkaline phosphatase; PT, prothrombin time; INT, international normalized ratio; GGT, gamma glutamyl transferase; CRP, C-reactive protein

* Histological findings were analyzed based on NAFLD activity scores developed by NASH clinical research network.

**Supplementary Table 2.** The main KEGG pathways of the screened eight miRNAs

| KEGG Term | Total genes of the term | Union targets in the term | Union miRNAs in the term | Score |
| --- | --- | --- | --- | --- |
| Focal adhesion | 199 | 47 | 5 | 2.700 |
| Pathway in cancer | 325 | 70 | 5 | 2.698 |
| Small-cell lung cancer | 84 | 26 | 5 | 2.253 |
| MAPK signaling pathway | 272 | 49 | 5 | 2.137 |
| Amoebiasis | 105 | 32 | 4 | 2.106 |
| Regulation of actin cytoskeleton | 213 | 40 | 5 | 2.006 |
| ECM receptor interaction | 84 | 23 | 4 | 1.887 |
| TGF-β signaling pathway | 84 | 19 | 4 | 1.867 |
| Wnt signaling pathway | 150 | 31 | 5 | 1.824 |
| Melanoma | 71 | 18 | 5 | 1.652 |

KEGG, Kyoto encyclopedia of genes and genomes; MAPK, mitogen-activated protein kinase; ECM, extracellular matrix; TGF, transforming growth factor

**Supplementary Table 3.** Primers used in quantitative real-time PCR

| Name | Sequence (5′-3′) |
| --- | --- |
| miRNA |  |
| miR-21-5p | UAGCUUAUCAGACUGAUGUUGA |
| miR-151a-3p | \| CUAGACUGAAGCUCCUUGAGG \| \| --- \| |
| miR-192-5p | CUGACCUAUGAAUUGACAGCC |
| miR-4449 | CGUCCCGGGGCUGCGCGAGGCA |
| U6 | GTGCTCGCTTCGGCAGCACATATACTAAAATTGGAACGATACAGAGAAGATTAGCATGGCCCCTGCGCAAGGATGACACGCAAATTCGTGAAGCGTTCCATATTTT |
